# Native Endophytic *Pseudomonas putida* as a Biocontrol Agent against Common Bean Rust Caused by *Uromyces appendiculatus*

**DOI:** 10.3390/jof7090745

**Published:** 2021-09-10

**Authors:** Kamal A. M. Abo-Elyousr, Ismail R. Abdel-Rahim, Najeeb M. Almasoudi, Sameera A. Alghamdi

**Affiliations:** 1 Department of Arid Land Agriculture, Faculty of Meteorology, Environment and Arid Land Agriculture, King Abdulaziz University, Jeddah 80208, Saudi Arabia; nalmasoudi@kau.edu.sa; 2 Botany and Microbiology Department, Faculty of Science, Assiut University, Assiut 71516, Egypt; ismailramadan@aun.edu.eg; 3 Department of Biological Sciences, Faculty of Sciences, King Abdulaziz University, Jeddah 21551, Saudi Arabia; saalghamdi17@gmail.com

**Keywords:** common bean, cell wall degrading enzymes, endophytes, *Pseudomonas putida*, *Uromyces appendiculatus*, rust disease

## Abstract

This study aimed to evaluate the efficacy of endophytic bacterium to control common bean rust disease under greenhouse conditions. Endophytic bacterium *Pseudomonas putida* ASU15 was isolated from fresh asymptomatic common bean, identified using biochemical and molecular characteristics. In vitro, the inhibitory effect of different concentrations of *P. putida* (1 × 10^4^, 1 × 10^5^ and 1 × 10^6^), as well as fungicide ortiva (0.01%) on uredospores germination of *Uromyces appendiculatus* were tested using water agar medium. The concentration showing the highest reduction of uredospores germination was at 1 × 10^6^, while there was complete inhibition of uredospores germination associated with using ortiva. Scanning electron microscope exhibited the ability of *P. putida* cells to attack the cell wall of the fungal uredospores germ tubes of *U. appendiculatus*, causing obvious cell wall breakdown. The activities of chitinase, lipase, and protease produced by *P. putida* ASU15, in vitro, were evaluated spectrophotometrically. Chitinolytic, proteolytic, and lipolytic activities were exhibited, contributing 55.26, 3.87, and 26.12 U/mL, respectively. Under greenhouse conditions, treated plants with *P. putida* ASU15 (two days before pathogen inoculation or at the same time of pathogen inoculation) or fungicide reduced the disease severity, compared to the control. Applying *P. putida* ASU15 at the same time of pathogen inoculation showed reduction in disease severity (69.9%), higher than application before pathogen inoculation (54.9%). This study is considered the first report that demonstrates the mycoparasitic strategy of *P. putida* for controlling *U. appendiculatus*. In conclusion, our results revealed that *P. putida* ASU15 affords a significant disease reduction that may be attributed to direct suppression of pathogen spores germination.

## 1. Introduction

Common bean (*Phaseolus vulgaris* L.) is one of the most important vegetable food legumes in Egypt for local consumption and exportation [1]. Bean rust, incited by *Uromyces appendiculatus* (Pers. Ex Pers.), is one of the most destructive, yield-limiting, worldwide diseases of common bean [2]. Economically, it causes yield losses ranging from 25 to 100% in susceptible cultivars [3,4]. Interestingly, it has been reported as an economical threat to the dry beans in South Africa, producing 100% losses for rust-susceptible varieties [5]. Indeed, bean rust was mainly managed using chemical fungicides [6]. The use of fungicides had been practiced and its success depends mainly on the high repetition of applications; however, fungicides were recently restricted in many countries for use against bean rust due to their adverse effects on non-target species, environmental pollution, and development of fungicide resistant strains, in addition to the harmful aspects they cause to humans and climate [7]. On the other hand, bean rust was sometimes controlled based on host resistance, but resistance has not been long-lasting due to the pathogen’s high genetic diversity and ability to evolve new pathogen strains. Actually, the use of plant resistance is not only harmless to the environment but also an economically sound strategy, compared to chemical control. However, the wide variability of *U. appendiculatus* represents an obstacle to breeders aiming at the development of common bean cultivars with durable resistance to rust [8].

Biological control of rust on bean has been previously explored as an effective approach for disease management [9]. It has been reported as a nature-friendly alternative method due to their ability to antagonize the pathogen by different modes of action and to effectively colonize distinct plant habitats [10,11]. Endophytic bacteria have been reported as potent biological control agents of several plant pathogens [12]. Endophytic bacteria have been defined as those that colonize the internal tissue of the plant, showing no external sign of infection or negative effect on their host [13]. Endophytes have been isolated from various parts of the plants, including the leaves, flowers, stems roots, seeds, and fruits [14]. Some of these bacteria are beneficial for their hosts; they can also accelerate seedling emergence, promote plant establishment under adverse conditions [15], and enhance plant growth [16]. Bacterial endophytes have been shown to prevent disease development [17]. Endophytic bacteria, as plant growth promoters, assist in the uptake of essential nutrients [9], and produce essential phytohormones [18]. On the other hand, these bacteria support plant defense against phytopathogens by developing antibiotics [10], producing hydrogen cyanide [19], competing for nutrients with phytopathogens [20], and causing systemic resistance in the host [21].

*Pseudomonas* is considered one of the most characterized biocontrol plant growth-promoting bacteria [22]. Moreover, many species of *Pseudomonas* have several advantages for use as biological control agents. *Pseudomonas putida* has been reported as an effective biocontrol agent against several plant pathogens such as *Rhizoctonia solani* in cucumber [23], *Fusarium oxysporum* f.sp. *radicis-lycopersici* in tomato [24,25], *R. solani* in potato [26], *Sclerotinia sclerotiorum* in lettuce [25], *Pectobacterium atrosepticum* in potato [25], and *Ralstonia solanacearum* in tomato [27]. Indeed, most of the previous studies attributed potentiality of the diseases management by *P. putida* to antibiotics production, competition on nutrients and space, and induction of host systemic resistance.

To our knowledge, this is the first report of direct mycoparasitic potentiality of *Pseudomonas putida* as a mode of action for controlling *Uromyces appendiculatus*. Thus, the present study aimed to: (1) isolate and identify endophytic bacteria *P. putida* from common bean plants and (2) evaluate the efficacy of endophytic bacteria for controlling bean rust under greenhouse conditions.

## 2. Materials and Methods

### 2.1. Source of Uromyces appendiculatus and Spores Collection and Preservation

The uredospores of *U. appendiculatus* were obtained from naturally infected plants that were cultivated in El-Menia Governorate, Egypt. For multiplication of the amount of spores, the obtained uredospores were used to re-infect the plants growing under greenhouse conditions at Assiut University. Two healthy bean seeds cv. Giza 6 were sown in plastic pots 15 cm in diameter (1.4 kg soil), filled with a sterilized soil. The pots were kept in the greenhouse at temperatures ranging between 20 and 25 °C and then the plants were fertilized when needed. The growing terminal bud of each plant was removed above the fourth or fifth leaf, to restrict the indeterminate growth of the cultivar, and to facilitate the handling of the plants [28]. Plants aged 25-days old were artificially inoculated by uredospores. To prepare the inoculum, uredospores were suspended in sterile distilled water and mixed with agar (0.1 g/L) before inoculation. The inoculation was done by spraying the plants with a suspension of uredospores with an atomizer (complete leaf coverage). The inoculated plants were kept in darkness for 48 h. Meanwhile, mature uredospores were collected from the leaves on aluminum foil and dried, then transferred to micro-tubes and stored under freezing conditions, for 15 days, until the following experiments.

### 2.2. Isolation of Endophytic Strain ASU15

Strain ASU15 was isolated, as endophytic bacteria, from fresh asymptomatic healthy leaves of common bean cultivated in Assiut Governorate (Egypt). The leaves samples were collected in sterilized polyethylene bags and immediately transferred to a microbiological laboratory. Bacterial isolation was carried out using a surface sterilization protocol described by Li et al. [29] and Abdelshafy et al. [30]. The samples of leaves were thoroughly washed under tap water and subsequently sterile distilled water to remove adhering debris and epiphytic microorganisms. The leaves were cut into small segments by using sterile scissors. Thereafter, the leaf segments were surface sterilized, under a laminar airflow cabinet, by soaking in 70% alcohol for 30 s with shaking, followed by immersion in a 2% aqueous solution of sodium hypochlorite for 2 min. Then, they were rinsed with sterilized distilled water 3 times (for removing the residues of the chemical agents applied in the surface sterilization) and further dried in sterilized paper. One gram of the surface-sterilized leaf segments was crushed and macerated under sterile conditions in a sterilized mortar and 9 mL of sterile sodium phosphate buffered saline was added. The tissue juice was collected in sterile polypropylene tube, homogenized by vortex for 2 min, and then centrifuged at 8000 rpm for 5 min. The supernatant was collected, serially diluted up to 10^−4^ using sterile 10 mM sodium phosphate buffer, pH 7. Then, one milliliter of the finally diluted solution of intercellular fluid of leave tissue was plated on nutrient agar plates in triplicate and kept in an incubator at 28 °C for 72 h. The growing bacterial colonies were streaked, purified, and identified.

### 2.3. Phenotypic Characterization of Endophytic pseudomonas putida Strain ASU15

Phenotypic characterization of the endophytic bacterium included cultural, morphological, microscopic, biochemical, and physiological quantifications following the classical tests described in Bergey’s Manual of Systematic Bacteriology [31]. Circumstantially, we evaluated colony morphology, pigmentation, cell morphology, mobility, Gram staining, utilization of carbon sources, enzyme activity, and growth on different pH values and salinity.

### 2.4. Genotypic Identification of Endophytic pseudomonas putida Strain ASU15

Bacterial DNA extraction was executed applying SDS/CTAB lysis and phenol/chloroform extraction technique. The extracted DNA was dissolved in 20 mL TE buffer and then used as a template for PCR. The universal primers set of 27F (5′-CAGAGTTTGATCCTGGCT-3′) and 1492R (5′-AGGAGGTGATCCAGCCGCA-3′) was selected for the amplification of 16S rRNA gene. The amplification process was carried out in a 25 µL reaction volume including 10–50 ng DNA template, 0.4 µM of each primer, 0.75 U EF-Taq DNA polymerase, 0.2 µM of each dNTP, and 1EF-Taq reaction buffer. The kits, enzymes, and chemical ingredients used in DNA extraction, purification, and amplification were manufactured by SolGent Company (Daejeon, Korea). The thermocycling conditions of PCR were operated as the following: 15 min at 95 °C for initial denaturation step, followed by 20 s at 95 °C for 35 cycles of denaturation, 40 s at 50 °C for annealing, 1.5 min at 72 °C for extension and then 5 min at 72 °C for a final extension step. For separation of the PCR product, gel electrophoresis (1.5% agarose having ethidium bromide plus a 0.5 Tris-acetate-EDTA (TAE) buffer) was applied. Moreover, the PCR product was visualized by a UV illuminator and then purified using a PCR purification kit. Sequencing of the PCR product was carried out using an ABI-Big Dye Terminator v3.1 cycle sequencing kit and an ABI 3730XL DNA analyzer (Applied Biosystems, Foster City, CA, USA). The obtained sequence of the 16S rRNA was analyzed using BLAST search program at the NCBI website: http://blast.ncbi.nlm.nih.gov/Blast.cgi (accessed on 19 October 2020). On comparison to 16S rRNA gene sequences of many standard strains from GenBank, the alignment was estimated using the multiple sequence alignment program CLUSTALW. Moreover, the molecular phylogenetic tree was constructed using MegAlign (ver. 5.01).

### 2.5. Determination of Suppressive Impact of Pseudomonas putida ASU15 on Uredospores Germination

Under light microscope, determination of the inhibitory impact of *P. putida* strain ASU15 on germination of uredospores of *U. appendiculatus* was carried out according to methods described by Li et al. [32]. Fresh uredospores (150 spores) were uniformly spread on the surface of water agar with 100 µL of *P. putida* strain ASU15 at different concentrations of 1 × 10^4^, 1 × 10^5^, and 1 × 10^6^ CFU mL^−1^. For each treatment, germination of spores in five visual fields was examined under a light microscope. Examination of the germination of uredospores on water agar without bacterial treatment was used as control, while the treatment with 100 µL of fungicide Ortiva 0.01% was used as the referenced treatment. All treatments were incubated at 19 °C for 6 h and then germination of uredospores was examined via a light microscope. The experiment was a completely randomized design with three replicates for each treatment, and then the experiment was repeated twice.

### 2.6. Scanning Electron Microscopy (SEM) Analysis

The potentiality of *P. putida* strain ASU15 to attack germinated uredospores of *Uromyces appendiculatus* was investigated using SEM that was available at the electron microscope unit, Assiut University. The samples of uredospores treated with *P. putida* strain ASU15 were fixed in 4% cold gautaraldehyde and then rinsed using C_2_H_6_AsNaO_2_ buffer. The prepared specimens were subsequently dehydrated by applying a gradual increase in the rate of ethanol, dried in critical point drainer by liquid CO_2_, and then cemented on the metallic block. The specimens were uniformly gold coated at a thickness of 15 nm using gold splutter apparatus. Eventually, the specimens were examined and photographed using JSM 5400 LV Scanning Electron Microscope (JEOL Ltd., Tokyo, Japan). SEM was operated at an acceleration voltage of 15 kV with magnification range 1000×–3500× and a 10 mm working distance under low vacuum.

### 2.7. Evaluation of Extracellular Enzymatic Activities

#### 2.7.1. Chitinase Activity

Chitinolytic activity was essayed using the technique described by Chen and Lee [33]. *P. putida* strain ASU15 was firstly grown for 48 h at 25 °C on a chitin-containing medium. This medium included (g/L) 6.9 NaH_2_PO_4_, 0.3 MgSO_4_·7H_2_O, 1.4 (NH_4_)_2_SO_4_, 10 peptone, 2.0 KH_2_PO_4_, and 1.0 colloidal chitin, whereby chitin was prepared by the 85% H_3_PO_4_ acid hydrolysis technique [34]. In a test tube, the reaction mixture (0.5 mL of culture filtrate and 1.0 mL of the prepared colloidal chitin) was incubated at 30 °C for 60 min. Then the reaction was terminated using 1.0 mL potassium sodium tartrate reagent and 1.0 mL of dinitrosalicylic acid. Spectrophotometrically, the liberated *N*-acetylglucosamine (NAG) units were measured at 540 nm as an indicator of chitinase activity [35]. Chitinase activity unit (U/mL) point out to the amount of enzyme required to produce 1 µmol of NAG min^−1^. Three replicates were carried out.

#### 2.7.2. Protease Activity

Inocula of *P. putida* strain ASU15 were aseptically transferred into Erlenmeyer flasks (250 mL) including 50 mL of sterilized Czapek-Dox medium provided with casein (50 mg) as a substrate for protease production [36]. Enzyme activity was essayed using the technique reported by Folin and Ciocalteu [37]. The test tube containing the reaction mixture (1 mL of crude enzyme and 5 mL casein) was vortexed and incubated for 10 min at 30 °C. The reaction was terminated using 5 mL trichloroacetic acid. Thereafter, 5 mL of Na_2_CO_3_ and 1 mL of Folin-Ciocalteu’s reagent were added. Spectrophotometrically, the absorbance was determined at 660 nm. The liberated amino acids were calculated using the standard curve of tyrosine. Protease activity unit (U/mL) was defined as the amount of enzyme required to liberate 1 μmol of tyrosine min^−1^. Protease activity was determined three times.

#### 2.7.3. Lipase Activity

*P. putida* strain ASU15 was inoculated into Erlenmeyer conical flasks containing 100 mL of lipase production medium at 30 °C under agitation (120 rpm) for 2 days. Lipase production medium containing (g/L) NH_4_NO_3_, 1; ZnSO_4_, 0.005; K_2_HPO_4_, 1.5; MgSO_4_, 0.025; FeSO_4_, 0.015; CaCl_2_, 0.025 and amended with 1 mL Tween 80 [38]. The bacterial biomass was eliminated using centrifugation at 80,000 rpm for 30 min under cooling (4 °C) and therefore the supernatants included the crude enzyme used for determination of lipase activity. Lipase activity was assayed using p-nitrophenylpalmitate (pNPP), as reported by Prazeres et al. [39]. Illustratively, a mixture of 1.0 mL of pNPP, 0.5 mL Triss buffer (100 mM, pH 7) and 1.0 mL crude enzyme was transferred into a tube and then the volume was finalized into 3 mL by sterilized distilled water. The mixture was incubated at 30 °C for 30 min. The reaction was terminated by adding isopropanol (0.2 mL). Spectrophotometrically, lipase activity was evaluated at 410 nm. Lipase activity unit (U/mL) represents μmol pNPP min^−1^ liberated by one milliliter of the bacterial crude enzyme under standard assay conditions. The result was represented by three replicates.

#### 2.7.4. Protein Assay

In order to calculate the specific activity, the concentration of extracellular protein that originated in the crude enzyme was determined [40]. The concentration of protein was expressed as mg/mL. A standard curve was carried out using bovine serum albumin. Three replicates were carried out. Eventually, the enzymatic specific activity was represented as μmol liberated monomers per mg protein per min.

### 2.8. Efficacy of Bacterium Isolate as Spraying Treatments for Controlling Common Bean Rust Disease under Greenhouse Conditions

#### 2.8.1. Inoculum Preparation, Inoculation Methods, and Evaluation of Disease Severity

The preserved uredospores were suspended in distilled water, followed by adding agar (0.1 g/L) and then thoroughly mixing before inoculation of the plants [41]. The concentration of spores was measured using a hemocytometer and adjusted to the desired concentration (1 × 10^6^ spores/mL) by the dilution method, as mentioned by Lopes and Berger [28]. Two healthy bean seeds Giz 6 cultivar were sown in each pot and after 15 days, the plants were inoculated with uredospores suspension, as mentioned above. Inoculation was done by spraying using an atomizer, whereas the plant leaves were completely covered with the spore suspension of *U. appendiculatus*. Plants were placed in a dark highly humid chamber (humidity > 90%, 22 °C). Plants sprayed only with water were used as control (healthy plants). Each treatment contained three replicates (three pots/treatment).

Disease incidence on the primary leaves of each plant was estimated. The disease severity was measured after 12–15 days of inoculation, according to the scale adopted for new procedures of the international classification [3]. This scale categorized the infection into six degrees: (1) no pustules; (2) necrotic spots without sporulation; (3) sporulating pustules with a diameter of <300 μm; (4) sporulating pustules with a diameter ranging between 300–499 μm; (5) sporulating pustules with a diameter ranging between 500–800 μm; and (6) sporulating pustules with a diameter of >800 μm.

#### 2.8.2. Determination of Suppressive Impact of *P. putida ASU15* on Incidence of Common Bean Rust

Under greenhouse conditions, the effect of spraying *P. putida* ASU15 and fungicide 0.01% Ortiva on incidence of common bean rust was evaluated. The bacterial suspension of 10^6^ CFU/mL was applied at two different times (two days before pathogen inoculation or at the same time of pathogen inoculation). After 15 days of pathogen inoculation, numbers and sizes of the pustules were determined and subsequently disease severity was calculated. The control plants were sprayed only with the pathogen. Three replicates were used for each treatment.

### 2.9. Statistical Analysis

The results were statistically analyzed via one-way ANOVA on the SPSS 10.0 (SPSS, Chicago, IL, USA) software program. On the other hand, results of the disease incidence under greenhouse conditions were analyzed based on two-way ANOVA. The mean as well as standard errors were computed for three replicates. Means were compared by Duncan’s multiple tests and the statistical significance was determined at the 5% level.

## 3. Results

### 3.1. Source of Uromyces appendiculatus

**Figure 1:**
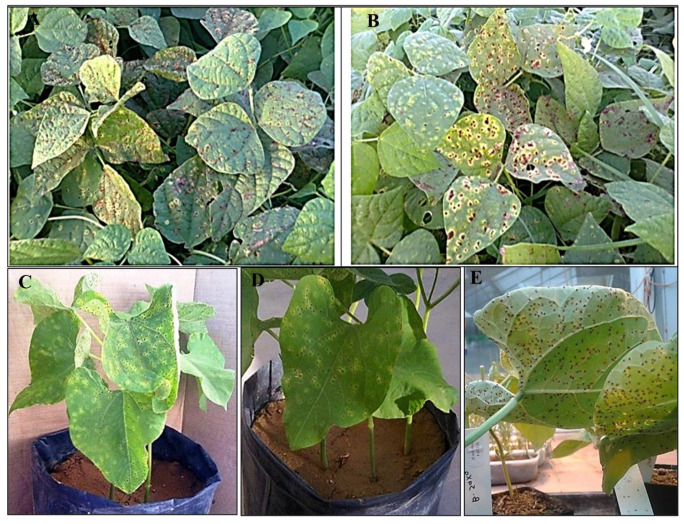
Common bean rust disease caused by *Uromyces appendiculatus*. (**A**,**B**) Naturally infected plants showing the symptoms of the rust disease; (**C**–**E**) Pathogenicity test exhibiting the ability of uredospores to re-infect the plants under greenhouse conditions. Developing of uredial lesions on the upper (**C**,**D**) and lower epidermis (**E**) of bean leaves.

The uredospores of *U. appendiculatus* were obtained from heavily infected bean plants in El-Menia Governorate, Egypt. Interestingly, rust-infected common bean plants in the field showed typical symptoms of the disease (Figure 1). Numerous reddish brown circular pustules of 1–2 mm in diameter, representing uredial lesions, develop on the upper and lower sides of bean leaves. Moreover, infected tissues of the leaves surrounding single large or small groups of uredial lesions turn yellow. The pustules rupture and liberate reddish brown uredospores. We collected the uredospores from uredial lesions of the naturally infected bean leaves and used them to re-infect bean plants cultivated in the greenhouse of Assiut University. The pathogenicity test exhibited the ability of *U. appendiculatus* spores to aggressively infect the plants, producing intensive symptoms of the disease (Figure 1).

### 3.2. Identification of Endophytic Bacterium

In this study, *Pseudomonas putida* strain ASU15 was isolated as endophytic bacterium, from healthy leaves of common bean cultivated in Assiut Governorate (Egypt). The bacterial isolate was preliminarily identified at the level of genus *Pseudomonas*, depending on colony morphology, microscopic characteristics, as well as biochemical and physiological activities (Table 1). The bacterial colony was entire, circular, convex, and translucent with smooth surface, fluorescent, yellowish and 2–3 mm diameter on the isolation medium at 28 °C after 48 h incubation. The bacterial cells were aerobic (unable to ferment glucose), Gram negative, single, motile small rods, 0.6–0.8 1.2–1.8 µm. It was positive for catalase, gelatin hydrolysis, cytochrome oxidase, arabinose, dextrose, fructose, galactose, mannitol, mannose, indole, casein, xylose and utilization of glucose, orgnic acids (citric, galacturonic, gluconic, glucuronic, capric acids), and indole production. On the other hand, it was negative for starch and H_2_S production, cellobiose, inositol, inulin, lactose, maltose, raffinose, rhamnose and sorbitol (Table 1).

**Figure 2:**
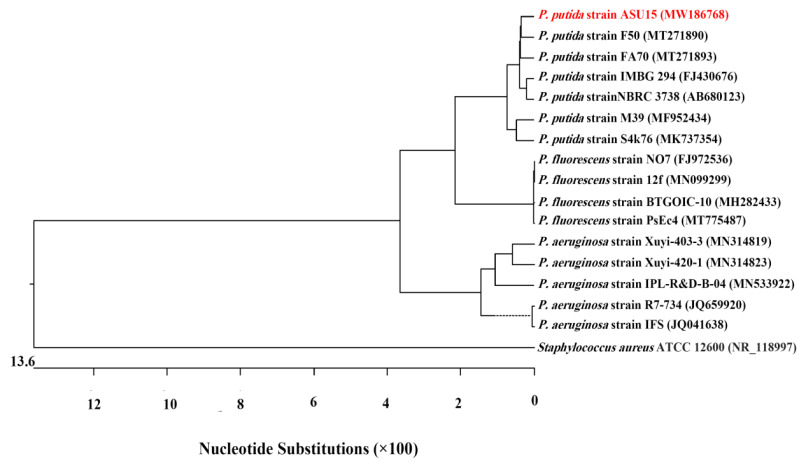
Molecular phylogenetic tree of 16S rRNA gene sequence of *Pseudomonas putida* strain ASU15 (red font) with related referential bacterial strains in GenBank database were constructed by the Neighbor-Joining method using MegAlign software (ver. 5.01). *Staphylococcus aureus* strain ATCC 12,600 (NR_118997) was used as an out-group. Accession numbers of the National Center for Biotechnology Information (NCBI) database of each strain are given in brackets.

The results of 16S rRNA analysis and gene sequences (1061 base pair) of our indigenous bacterium *Pseudomonas* strain ASU15 showed 99.81% similarity to *P. putida* strain F50 (MT271890). The 16S rRNA sequence of *P. putida* strain ASU15 was deposited into GenBank under accession number MW186768. A phylogenetic tree was estimated with other closely related neighboring sequences of different referential strains at GenBank (Figure 2).

### 3.3. Inhibitory Impact of P. putida ASU15 on Germination Rate of Uredospores

The results presented in Table 2 revealed that the applying of *P. putida* ASU15 significantly inhibited the germination rate of urediniospore. Moreover, the rate of uredospores germination was reduced with increase in bacterial concentration. The germination rates of the urediniospore treated with *P. putida* ASU15 1 × 10^4^, 1 × 10^5^, and 1 × 10^6^ CFU mL^−1^ were 16.00, 11.33, and 4.67%, respectively, corresponding to inhibition rates of 83.89, 88.59, and 95.30%, respectively (Table 2). The rate of uredospores germination on control was 99.33%, while the use of fungicide Ortiva (0.01%) showed complete inhibition of uredospores germination.

### 3.4. Scanning Electron Microscopy (SEM) Analysis

**Figure 3:**
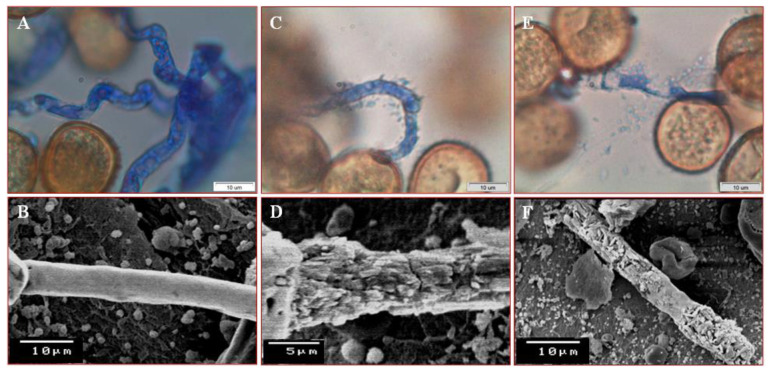
Light and scanning electron micrographs showing the inhibitory impact of *Pseudomonas putida* strain ASU15on uredospores of *Uromyces appendiculatus.* (**A**,**B**) Normal germinating uredospores (control) showing vigor germ tube; (**C**,**D**) *P. putida* cells attack the germ tube; (**E**,**F**) Degradation of cell wall of the germ tube by mycoparasitic activity of *P. putida* and then release of the cytoplasmic contents from a ruptured germ tube.

Figure 3 exhibited an illustrative picture of the establishment of antagonistic activity of *P. putida* on germ tube of uredospores of *U. appendiculatus* (Figure 3). Preliminary, *P. putida* cells were in close contact with the germ tube’s cell wall of uredospores for an attack. Lytic enzymes produced by *P. putida* degraded the cell wall of the germ tubes. Subsequently, a ruptured germ tube allowed to release the internal cytoplasmic contents. Thereafter, *P. putida* caused distinct deformation of the germ tube, compared with the non-treated one.

### 3.5. Extracellular Enzymatic Activity

The results presented in Table 3 indicated that the endophytic bacterium *P. putida* strain ASU15 has the potential to secrete various enzymes that play an important role in the decaying and distortion of the fungal pathogen. The activities of chitinolytic, lipolytic, and proteolytic enzymes produced by *P. putida* strain ASU15 were evaluated spectrophotometrically in vitro. The *P. putida* strain ASU15 exhibited chitinase activity 55.26 U/mL with a specific activity up to 8.96 U/mg proteins. The proteolytic activity was 3.87 U/mL, corresponding to a specific activity of 8.41 U/mg proteins, while lipase activity amounted to 26.12 U/mL, which was equivalent to a specific activity of 9.20 U/mg protein (Table 3).

Units of the enzymatic activity (U) were expressed as follows: μmol *N*-acetylglucosamine min^−1^ for chitinase, μmol pNPP min^−1^ for lipase, and μmol of tyrosine min^−1^ for protease.

### 3.6. Suppressive Impact of P. putida ASU15 on Incidence of Common Bean Rust

Table 4 presented the inhibitory effect of *Pseudomonas putida* strain ASU15, as a foliar treatment, on disease severity of common bean rust under greenhouse conditions. Application of *P. putida* ASU1 (10^6^) at different times (two days before the pathogen inoculation or at the same time of the pathogen inoculation) as well as fungicide reduced disease severity, compared to the control treatment. The highest reduction was achieved by the fungicide (Ortiva 0.01%), contributing 93%. Treated plants with *P. putida* strain ASU15, at the same time of the pathogen inoculation, showed reduction in disease severity (69.9%), higher than application two days before pathogen inoculation (54.9).

## 4. Discussion

In the current study, *Pseudomonas* sp. was identified, as the endophytic bacterium isolated from healthy bean plants, based on colony morphology, microscopic characteristics, and biochemical tests. Then, identification was confirmed at the molecular level using 16S rRNA gene sequences. Genotypic identification of the *Pseudomonas* strain ASU15 was related to *P. putida* strain F50 (MT271890). This bacterium could be isolated as endophytic bacteria from the roots of plant species such as lemon and cotton [42,43], beet [44], and wheat cultivars [45]. Endophytic bacteria *Bacillus* sp. and *Pseudomonas* sp can be isolated from different plant species [46]. The phylogenetic analyses were carried out based on 16S rRNA gene sequences, which are considered as core ‘housekeeping’ genes of bacteria and used as key evidence for bacterial identification [47].

In vitro, the obtained results showed that *P. putida* ASU15 was able to inhibit the germination rate of urediniospore of *U. appendiculatus* by increasing the bacterial concentration. The highest reduction of urediniospore was at 1 × 10^6^ CFU mL^−1^. These results are in accordance with those obtained by Abeysinghe [48], who mentioned that the use of rhizobcateria isolated from bean plant can reduce the germination of *U. appendiculatu. Pseudomonas putida* P286 reduced the urediniospore germination of *Hemileia vastatrix* [49].

Light and scanning electron microscope examinations of uredospores with antagonistic bacterium exhibited abnormalities, lysis, collapse, and shrinking as a direct effect of the *P. putida*. Our results mentioned that the pathogen was inhibited during spore germination. Only free water and leaf topography are linked to the pathogen’s pre-penetration structure [50]. Based on this, the *P. putida* P286 isolate generated an antifungal compound that prevented the germination of uredospores [49]. The *Pseudomonas* species are recognized plant disease biocontrol agents. Production of antimicrobial compounds is one of the mechanisms associated with the antagonistic effect of these species [51]. Rajendran and Samiyappan [52] noted that using endophytic bacteria as whole cells can suppress some plant pathogenic fungi due to antimicrobial compounds that cause structural architect changes and lysis of mycelia [53].

In this study, the tested bacterial endophytes *P. putida* strain ASU15 produced chitinase, lipase, and protease. Endophytic microbes produce a variety of hydrolytic enzymes such as chitinase, lipase, and protease [54] that promote plant growth and preserve it from phytopathogens [55]. Hydrolytic enzymes can enhance plant growth by hydrolyzing phytopathogen cell walls [56]. Chitinase activities can hydrolyze chitin in the fungal cell wall, which leads to control of phytopathogenic fungi and promotes plant growth [57]. Hallmann Hallmann et al. [58] mentioned that the extracellular enzymatic activities of endophytic microbes could improve the induced systematic resistance in the plant.

Under greenhouse conditions, applying *P. putida* ASU15 two days or at the same time of inoculation with *U. appendiculatus* reduced the disease severity of bean rust disease compared with the control. Treating plants with bacteria at the same time of inoculation showed higher reduction in disease severity than application before inoculation. Endophytic bacteria have been used to control a wide range of pathogens [59]. Fouda et al. [60] reported that the highest concentration of bacteria leads to an increase in disease control. The antifungal substance produced by the antagonist bacteria was responsible for disease control—there is a strong correlation between antagonist concentrations and disease control. Antibiotics and volatile organic antifungal and antiviral compounds are among the secondary metabolic products generated by *Pseudomonas* [37,49,50] that affect plant pathogens.

## 5. Conclusions

In conclusion, our results showed that *P. putida* (ASU15) afforded a strong reduction of spore germination of *U. appendiculatus* in vitro and a high reduction in the development of rust severity in vivo. Under greenhouse conditions, treatments with ASU15 and fungicide reduce disease severity compared to the control treatment—the highest reduction was achieved by Ortives, fungicide 0.01% 93%, followed by ASU15 54.9–69.9%.

## Figures and Tables

**Table 1:** Morphological characteristics, physiological, and biochemical tests for characterization and identification of the endophytic bacterium *Pseudomonas putida strain ASU15*.

Characteristics and Tests	Results	Characteristics and Tests	Results
Morphological characteristics			
Colony morphology	Circular, convex and translucent	Colony color	Yellowish
Colony surface	Smooth	Colony margin	Entire
Colony diameter	2–3 mm	Fluorescent	+
Cell shape	Short rods	Motility	+
Gram Stain	Gram-ve	Spore formation	-
The growth and utilization of different carbon sources			
Glucose	+	d-Galacturonic acid	+
Mannose	+	d-Gluconic acid	+
Fructose	+	d-Glucuronic acid	+
Galactose	+	Methyl Pyruvate	+
Maltose	-	l-Lactic acid	+
Raffinose	-	Citric acid	+
Xylose	+	d-Malic acid	-
Cellobiose	-	l-Malic acid	+
Sucrose	-	Acetoacetic acid	-
Lactose	-	Propionic acid	+
Melibiose	+	Acetic acid	+
l-arabinose	+	Formic acid	+
Rhamnose	-	Tween 40	+
Inulin	-	d-Serine	-
Dextrose	+	l-Serine	+
Inosine	+	l-Alanine	+
Gelatin	+	l-Arginine	+
Pectin	-	d-Aspartic acid	-
Sorbitol	-	l-Aspartic acid	+
Mannitol	+	l-Glutamic acid	+
d-Arabitol	-	l-Pyroglutamic acid	+
Glycerol	-	Inositol	-
Growth at pH 5	+	Sodium butyrate	+
Growth at pH 6	+	Sodium bromate	-
Growth at 1% NaCl	+	1% Sodium lactate	+
Growth at 4% NaCl	+		
Growth at 8% NaCl	-		
Physiological and enzymatic activities			
Glucose fermentation	-	Casein hydrolysis	+
Cellulase	-	Starch hydrolysis	-
Catalase	+	HCN production	-
Oxidase	+	H_2_S production	-
Urease	+	Nitrate reduction	+
Indole acetic acid	+		

**Table 2:** Inhibitory impact of *Pseudomonas putida* strain ASU15 on germination rate of uredospores of *Uromyces appendiculatus*.

Treatments (Concentration)	Germination Rate	% Inhibition
*P. putida* (10 × 10^3^ CFU mL^−1^)	16.00 ± 1.30 ^b^	83.89
*P. putida* (10 × 10^5^ CFU mL^−1^)	11.33 ± 1.60 ^c^	88.59
*P. putida* (10 × 10^6^ CFU mL^−1^)	4.67 ± 1.20 ^d^	95.30
Fungicide Ortiva (0.01%)	0.00 ± 0.00 ^e^	100.00
Control	99.33 ± 0.88 ^a^	

Numbers within column are means ± standard deviations of three replicates. Values within column that associate with different letters indicate significant differences (*p* ≤ 0.05) based on one-way ANOVA analysis. The data are the means of more than 150 spores in five visual fields under light microscope in each treatment with three replications.

**Table 3:** Evaluation of the enzymatic activities of endophytic bacterium *Pseudomonas putida* strain ASU15.

Extracellular Enzymes	Chitinase	Lipase	Protease
Enzymatic activity (U/mL)	55.26 ± 0.03	26.12 ± 0.02	3.87 ± 0.03
Protein (mg/mL)	6.17 ± 0.04	2.84 ± 0.02	0.46 ± 0.04
Specific activity (U/mg protein)	8.96 ± 0.20	9.20 ± 0.20	8.41 ± 0.12

**Table 4:** Inhibitory effect of *Pseudomonas putida* strain ASU15, as a foliar treatment, on disease severity of common bean rust under greenhouse conditions.

Treatments	Two Days before the Pathogen		At the Same Time of Pathogen Inoculation		Mean
	Inoculation				
	Severity	Reduction %	Severity	Reduction %	
*P. putida* ASU15	26.67 ^b^	69.9	21.67 ^b^	54.8	31.67 ^b^
Ortiva	5.00 ^c^	93	5.00 ^c^	92.9	5.00 ^c^
Control	70.83 ^a^	-	71.67 ^a^	-	70.00 ^a^

Values within the column that associate with different letters indicate significant differences (*p* ≤ 0.05) based on two-way ANOVA analysis.

## Data Availability

Not applicable.
